# Supplementary material for: The Feasibility of Host Transcriptome Profiling as a Diagnostic Tool for Microbial Etiology in Childhood Cancer Patients with Febrile Neutropenia
Source: Int J Mol Sci. 2020 Jul 26;21(15):5305. doi: 10.3390/ijms21155305 (PMC7432212; doi:10.3390/ijms21155305)
Supplement: Supplementary file 1 [file ijms-21-05305-s001.pdf]

Table S1. Differentially expressed genes between the bacterial etiology and the viral, unknown, and co-infection etiologies, respectively.

Gene symbol	*log2FC*	*p-value*	*Gene symbol*	*log2FC*	*p-value*	*Gene symbol*	*log2FC*	*p-value*
SLCO5A1	5,8896656	1,57478E-09	SLCO5A1	6,1587415	9,12933E-10	POSTN	8,048523	3,03927E-08
FST	5,1366186	2,19971E-06	DPYSL4	3,6333992	1,33097E-06	FST	7,9196506	5,471E-05
TSPEAR	4,0919952	6,80506E-05	CAMKMT	1,356763	1,78692E-05	SLCO5A1	5,790431	1,82729E-05
CTD-3088G3.8	2,8793552	5,81385E-06	ZNF586	1,1383559	2,06425E-05	KIAA1257	2,940124	1,86589E-06
KIAA1257	2,4798577	0,000119117	DCLRE1B	-1,4864261	1,88121E-05	H2AFX	-1,4775257	4,77639E-05
CA6	2,41042	0,000139183	CTSL	-2,3995886	1,41181E-05	NAPRT	-2,0763056	3,52586E-05
ZNF66	2,3477285	2,79219E-10	SIGLEC15	-7,8543515	4,42698E-06	MRPL23	-2,1757846	5,07783E-05
ZNF573	1,7819986	8,74292E-05				NT5DC2	-2,6081164	1,09585E-06
KLHDC1	1,7300003	0,000107439				FAM198B	-2,6709602	6,37533E-05
PPP1R12B	1,5797136	1,65034E-05				BMP8B	-2,7487576	1,40335E-05
KATNBL1	1,4200376	2,32179E-06				SAPCD2	-3,1609368	2,19634E-05
ZNF486	1,378842	6,7382E-07				IER5L	-3,1832097	3,8867E-05
RPS6KA5	1,3517255	4,2703E-06				S100A8	-3,5481675	8,38103E-05
CASS4	1,3050755	2,53825E-05				SIGLEC12	-3,5775585	4,17237E-07
LRRC69	1,3028059	7,24728E-05				FZD2	-3,6326587	2,60279E-05
ZNF546	1,2928909	0,000123802				CD34	-3,6811168	2,81667E-05
ZRANB3	1,2782532	7,8642E-05				FAM20A	-3,7577186	2,69022E-05
ZNF69	1,2658635	7,48973E-05				DNM1	-3,792977	8,73582E-06
CAMKMT	1,2523932	5,26608E-06				HP	-3,892731	3,61779E-06
ELF2	1,0250338	0,000111199				MS4A4A	-4,1025453	3,15669E-07
RPAP2	1,0147629	6,4236E-05				ARHGEF17	-4,1058087	2,48883E-05
SLC25A16	0,9688366	0,000108917				KCNK17	-4,1858115	2,04327E-06
TRMT10B	0,7061428	0,000116328				GPER1	-4,2422633	6,52907E-05
C6orf229	0,66102964	3,1434E-05				HOXA9	-4,26747	1,15508E-07
KCTD5	-1,0027279	3,65387E-05				CD177	-4,413553	6,87268E-06
HPS6	-1,2350525	7,6516E-05				LAMC1	-5,0807	1,85147E-05
SERTAD1	-1,3517268	8,49319E-05				CYTL1	-5,477478	1,07947E-05
CDK2AP2	-1,4476525	9,68162E-05				METTL7B	-5,6511292	5,2719E-06
ATP5D	-1,506319	0,000112126				CYYR1	-5,9321055	9,02069E-05
CLIC4	-1,5283251	0,000119197				NPR3	-6,579038	3,37611E-05
C1orf122	-1,6132164	9,22131E-05				CLECL1	-6,9611735	2,17754E-05
MRPL23	-1,7651284	9,98417E-06				SIGLEC15	-7,4829006	6,77941E-05

LENG9	-1,8437638	4,71629E-05
NMB	-1,965787	2,96382E-05
FAM109B	-1,9682145	0,000117635
SOWAHD	-2,018248	0,000120716
TROAP	-2,96692	0,000120525
SAPCD2	-2,9851506	4,50217E-05
MMP17	-3,0916808	3,09208E-06
FZD2	-3,2316787	1,71585E-05
MS4A4A	-4,0543814	0,000148481
COCH	-4,989459	4,37113E-05
LAMC1	-5,0651145	1,42048E-05
CD22	-5,290688	3,27971E-05
SMIM1	-5,3259816	0,000131609
CLECL1	-5,673439	0,000115497
SIGLEC15	-7,2758484	1,47149E-05
